# *Salmonella spvC* Gene Inhibits Autophagy of Host Cells and Suppresses NLRP3 as Well as NLRC4

**DOI:** 10.3389/fimmu.2021.639019

**Published:** 2021-07-14

**Authors:** Liting Zhou, Yuanyuan Li, Song Gao, Haibo Yuan, Lingli Zuo, Chaoyi Wu, Rui Huang, Shuyan Wu

**Affiliations:** Department of Medical Microbiology, School of Biology and Basic Medical Science, Medical College of Soochow University, Suzhou, China

**Keywords:** *Salmonella*, *spvC*, autophagy, NLRP3, NLRC4

## Abstract

*Salmonella spvC* gene, encoding a phosphothreonine lyase on host mitogen-activated protein kinases, facilitates systemic infection of *Salmonella* while the precise mechanisms remain elusive. Autophagy and pyroptosis dependent on the activation of inflammasomes, as parts of innate immune response, contribute to host defense against *Salmonella* infection. Recently, we reported that *spvC* could inhibit pyroptosis. To explore the effect of *spvC* on autophagy and the relationship between its function in pyroptosis and autophagy, infection models of macrophages J774A.1 and epithelial HeLa cells co-cultured with *Salmonella* Typhimurium wild type, *spvC* deletion, site-directed mutant which lacks phosphothreonine lyase activity, or complemented strain were established. The levels of LC3 turnover and Beclin 1 of J774A.1 cells were determined by western blot. Confocal laser scanning microscopy was used to visualize the autophagic flux after being transfected with mRFP-GFP-LC3 plasmid in HeLa cells. Results showed that SpvC inhibited autophagosome formation through its phosphothreonine lyase activity. Additionally, analysis of nucleotide-binding oligomerization domain, leucine-rich repeat and pyrin domain-containing 3 (NLRP3) and NLR with CARD domain-containing 4 (NLRC4) in J774A.1 cells indicated that *spvC* decreased the protein levels of NLRP3 and NLRC4, which were significantly changed by autophagy inhibitor Bafilomycin A1. Together, our observations reveal a novel mechanism of *spvC* in *Salmonella* pathogenesis and host inflammatory response *via* inhibiting autophagy and NLRP3 as well as NLRC4. These pathways and their subversion by diverse pathogen virulence determinants are expected to throw light on the design of anti-infective agents.

## Introduction

*Salmonella* is a facultative intracellular pathogen that causes a serious threat to global public health. Centers for Disease Control and Prevention estimates approximately 1.35 million infections, 26,500 hospitalizations, and 420 deaths caused by *Salmonella* in the United States every year (1). Among 2600 *Salmonella enterica* serovars, *Salmonella enterica* serovar typhimurium (*S*. Typhimurium) is one of the most common isolates causing infection with a broad range of hosts (2). Therefore, it will be clinically important to develop new strategies to control *S*. Typhimurium infection. In particular, pathogenesis of *S*. Typhimurium requires the action of multiple virulence factors. SpvB and SpvC, virulence factors encoded within the *Salmonella* plasmid virulence (*spv*) operon, are responsible for pathogenicity of *S*. Typhimurium (3). Previously we reported a novel contribution of *spvB* to *Salmonella* pathogenesis through interfering with intracellular iron homeostasis (4). *spvC*, another essential factor of *Salmonella* virulence determinant encoding phosphothreonine lyase, suppresses intestinal inflammation and aggravates systemic dissemination through mitogen-activated protein kinase (MAPK) signaling pathway (5, 6). However, the underlying mechanisms have been only partly illuminated.

Intracellular pathogens invade mammalian host cells in membrane bound vesicles called phagosomes. Of note, autophagy is a process whereby a double-membrane structure (autophagosome) engulfs unnecessary invading pathogens and delivers them to the lysosome for degradation. But the pathogens have developed several survival mechanisms to prevent this degradation event (7). *S*. Typhimurium, including its effectors, has evolved to block host signaling cascades or even create favorable conditions for self-replication and survival by virtue of autophagy through specific mechanisms, so as to resist the host defense (8, 9). It has been reported that *spvC* is responsible for the anti-inflammatory effect of *S*. Typhimurium to facilitate bacterial dissemination, and the host can eliminate intracellular bacteria by autophagy, which can inhibit the further spread of bacteria. In light of this, we hypothesize that autophagy is also involved in *spvC*-mediated infection while the precise mechanism remains obscure.

Innate immune recognition is initiated by pattern-recognition receptors (PRRs), of which nucleotide-binding domain and leucine-rich repeat receptors (NLRs) function in the recognition of danger signals introduced into the host cell cytosol. Nucleotide-binding oligomerization domain, leucine-rich repeat and pyrin domain-containing 3 (NLRP3) responds to a diverse range of stimuli, including pathogens, microbial toxins, etc. NLRP3 plays a pivotal role in regulating live-or-die cell-fate decisions (10, 11). Inhibition of the NLRP3 inflammasome using MCC950 enhances host protection against *B. cereus*-induced infection (12). Besides, NLR with CARD domain-containing 4 (NLRC4) in epithelium is sufficient to protect against *S*. Typhimurium invasion (13). Assembly of the NLRP3 and NLRC4 inflammasomes leads to caspase 1-dependent/independent release of the pro-inflammatory cytokines IL-1β and IL-18, as well as to gasdermin D-mediated pyroptosis (14, 15). NLRP3 recruited by NLRC4 had been considered distinct inflammasome scaffolds in response to *S*. Typhimurium infection (16). Moreover, NLRP3 could be activated by flagellin under conditions of suboptimal NAIP/NLRC4 activation in *S*. Typhimurium infected macrophages (17). Our recent research revealed that *spvC* inhibits NLRP3 and NLRC4-associated pyroptosis against *S*. Typhimurium (18). Reports showed autophagy machinery constitutes a key cellular monitoring system that prevents excessive NLRP3 inflammasome activation (19, 20). However, more thorough investigation is required to shed light on the fundamental mechanisms underlying autophagy regulated NLRP3 and NLRC4 mediated by *spvC*.

Herein, both macrophages J774A.1 and epithelial HeLa cells were co-cultured with *S*. Typhimurium wild type, *spvC* deletion, site-directed mutant which lacks phosphothreonine lyase activity or complemented strain. We report a novel contribution of *spvC* to *S*. Typhimurium pathogenesis through the inhibition of host autophagy *via* its phosphothreonine lyase activity which affects the protein level of NLRP3 and NLRC4.

## Materials and Methods

### Bacterial Strains and Culture Conditions

*S*. Typhimurium wild type strain (STM-WT) was kindly supplied by Professor Qian Yang (Nanjing Agricultural University, Nanjing, China). STM-WT, *spvC* deletion mutant (STM-*ΔspvC*), *spvC* site-directed mutant (STM-*ΔspvC*/p*spvC* K136A) which lacks phosphothreonine lyase activity and *spvC* complemented strain (STM-*ΔspvC*/p*spvC*) were grown to log phase at 37°C in Luria Bertani (LB, Hangwei, China) broth overnight. STM-*ΔspvC*/p*spvC* K136A and STM-*ΔspvC*/p*spvC* were cultured in the media with 100 μg/ml ampicillin (Sigma, USA).

### Construction of Mutant Strains

STM-*ΔspvC* was constructed with λRed recombination system basically as previously described (21) and the corresponding plasmids were gifts from Professor Daoguo Zhou (Purdue University, West Lafayette, USA). STM-*ΔspvC*/p*spvC* K136A mutation was constructed by overlap PCR. STM-*ΔspvC*/p*spvC* K136A and STM-*ΔspvC*/p*spvC* was complemented with STM-*ΔspvC* using pBAD/gIII expression system (22). *spvC* deletion mutant, site-directed mutant and complemented strain were identified by PCR and sequencing.

### Cell Culture

J774A.1 cells were purchased from the Procell Life Science & Technology Co.,Ltd. HeLa cells were acquired from National Collection of Authenticated Cell Cultures. Cells were routinely cultured in complete medium that Dulbecco’s modified Eagle medium (HyClone Laboratories, Logan, UT, USA) supplemented with 10% (v/v) fetal bovine serum (Biological Industries, Kibbutz Beit-Haemek, Israel) and in a humidified incubator containing 5% CO_2_ at 37°C.

### Bacterial Infection

J774A.1 cells (1×10^6^/well) and HeLa cells (5×10^5^/well) were seeded in 12-well plates. On the day of infection, *S*. Typhimurium were diluted 1:100 with LB broth to subculture for 3 h. Both STM-*ΔspvC*/p*spvC* K136A and STM-*ΔspvC*/p*spvC* were supplemented with 0.2% L-arabinose (Sigma, USA). Bacteria were then washed three times in PBS. The optical density of bacteria was determined by spectrophotometry at 600 nm with viable plate counts before infection. The bacterial suspension was subsequently added to cultured cells at the multiplicity of infection (MOI) described in the figure legends. Fresh medium containing amikacin (100 μg/ml, Sigma, Burlington, MA, USA) was added to kill the extracellular bacteria at 1 hour post infection (hpi). Afterwards, infected cells were washed and subsequently cultured in fresh medium containing amikacin (10 μg/ml) to limit extracellular replication of bacteria. PD0325901 (50 nM, Selleck, USA) functioned as an ERK inhibitor was added to the complete medium mentioned in *Cell Culture* section 24 h before infection. Cells were pretreated with Bafilomycin A1 (100 nM, Sigma, USA) 2 h before infection to inhibit autophagosome-lysosome fusion. At different time points following infection, cells were processed in the following ways.

### Western Blot Analysis

Proteins were extracted using RIPA buffer containing protease inhibitors and phosphatase inhibitors (Beyotime, China). Samples were homogenized on ice, centrifuged for supernatant at 12,000 g for 15 min at 4°C and heated to 100°C for 5 min. Protein extracts resuspended in sample loading buffer were separated by electrophoresis through 12% polyacrylamide gels and transferred to PVDF membranes (Millipore, USA). After blocking with 5% non-fat milk (Sangon Biotech Shanghai Co.,Ltd., China), membranes were incubated with primary antibodies anti-LC3 (4108S, CST, USA; 1: 1,000 dilution), anti-Beclin 1 (3738, CST, USA; 1: 1,000 dilution), anti-NLRP3 (15101S, CST, USA; 1: 1,000 dilution), anti-NLRC4 (ab201792, abcam, UK; 1: 1,000 dilution), anti-GAPDH (BA2913, Boster, China; 1: 1,000 dilution), anti-Tubulin (AF1216, Beyotime, China; 1: 1,000 dilution) and anti-Histone H3 (ab194681, abcam, UK; 1: 1,000 dilution) overnight at 4°C. Membranes were then washed and incubated with the horseradish peroxidase (HRP)-labeled goat anti-rabbit IgG (A0208, Beyotime, China; 1: 3,000 dilution) for 1 h at room temperature. Proteins were visualized using ECL luminescence reagent (Meilunbio, China). The gray-scale values of the bands were determined by Image J launcher broken symmetry software program (National Institutes of Health, Bethesda, MD, USA).

### Confocal Laser Scanning Microscopy

For immunofluorescence microscopy, HeLa cells transfected with mRFP-GFP-LC3 plasmid were used to establish the infection model. HeLa cells were collected at 2 hpi, bacteria and cell nucleus were labeled by Hoechst (33258, Beyotime, China; 1: 10,000 dilution). The samples were imaged under a confocal laser scanning microscopy (Fluoview FV1000, Olympus, Japan). GFP-LC3 and mRFP-LC3 punctate dots were counted in more than 100 cells and measured by Image J software program for quantification.

### Statistical Analysis

Statistical significance was determined by ANOVA for three or more groups. *P* < 0.05 was considered to be statistically significant.

## Results

### *spvC* Suppresses Autophagy and Increases Intracellular Survival of *Salmonella* Typhimurium in Macrophages at the Early Stage of Infection

**Figure 1:**
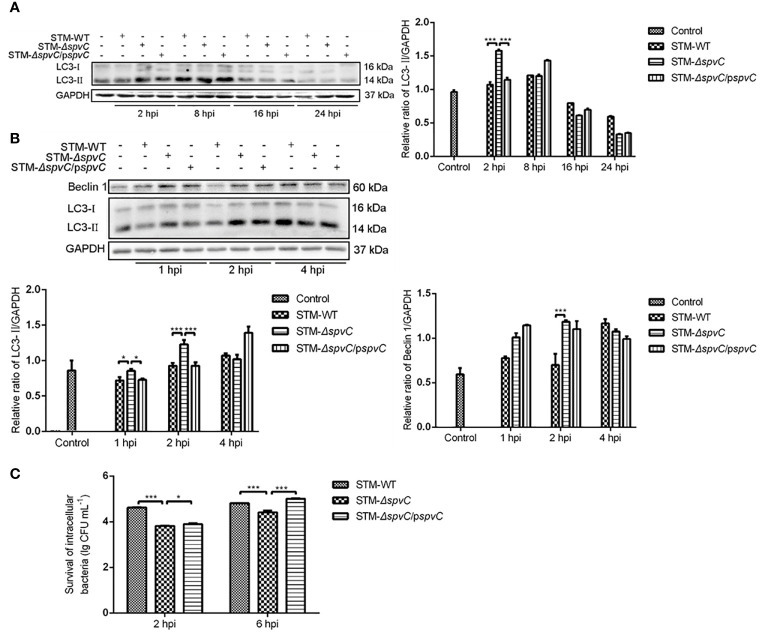
*SpvC* suppresses autophagy and increases intracellular survival of *Salmonella* Typhimurium in macrophages at the early stage of infection. **(A, B)** J774A.1 cells were infected with STM-WT, STM-*ΔspvC* or STM-*ΔspvC*/p*spvC* at an MOI of 10. Cell lysates were analysed by western blot with specific antibodies to LC3 and the control GAPDH at 2 hpi, 8 hpi, 16 hpi and 24 hpi **(A)**, to Beclin 1, LC3 and the control GAPDH at 1 hpi, 2 hpi and 4 hpi **(B)**. **(C)** Intracellular bacterial loads of J774A. 1 cells were assayed by colony forming units counting. Data were compared by ANOVA. Values are expressed as the means ± S.D., n = 3. Statistically significant differences are indicated. ***P < 0.001; *P < 0.05.

Our previous study revealed that *Salmonella* plasmid virulence gene *spvB* impair autophagic flux in infected macrophages for pathogen clearance (23). To determine the role of *spvC* on host cell autophagy, J774A.1 cells were collected to detect the expression of Microtubule associated protein light chain 3 (LC3) by western blot after being co-cultured with STM-WT, STM-*ΔspvC* or STM-*ΔspvC*/p*spvC* at 2 hpi, 8 hpi, 16 hpi and 24 hpi (**Figure 1A**). We found an increasing evidence of LC3-II in the early stage of infection (2 hpi and 8 hpi) compared with that in the late stage of infection (16 hpi and 24 hpi). At 2 hpi, higher level of LC3-II was found in STM-*ΔspvC* infected J774A.1 cells than those in STM-WT or STM-*ΔspvC*/p*spvC* infected cells. We next focused on the early stage of infection, and data confirmed that much more LC3-II and Beclin 1 were assessed in macrophages infected with STM-*ΔspvC* than in those infected with *S*. Typhimurium carrying *spvC* (**Figure 1B**). These data suggest that *spvC* suppresses autophagy in macrophages at the early stage of infection.

Autophagy is a cellular mechanism involving the degradation of cellular components or intracellular microbes through lysosomal machinery. We next examined the effect of the *Salmonella spvC* gene on intracellular bacterial loads. In agreement with our previous studies *in vivo* (18), macrophages J774A.1 infected with STM-*ΔspvC* showed significantly lower bacterial burden than those infected with STM-WT or STM-*ΔspvC*/p*spvC* since 2 hpi *in vitro* (**Figure 1C**). The aforementioned results suggest s*pvC* restricts elimination of pathogens in host cells which may be related to its contribution to autophagy, while the underlying mechanism remains elusive.

### *spvC* Inhibits the Formation of Autophagosomes in Host Cells During *Salmonella* Typhimurium Infection

**Figure 2:**
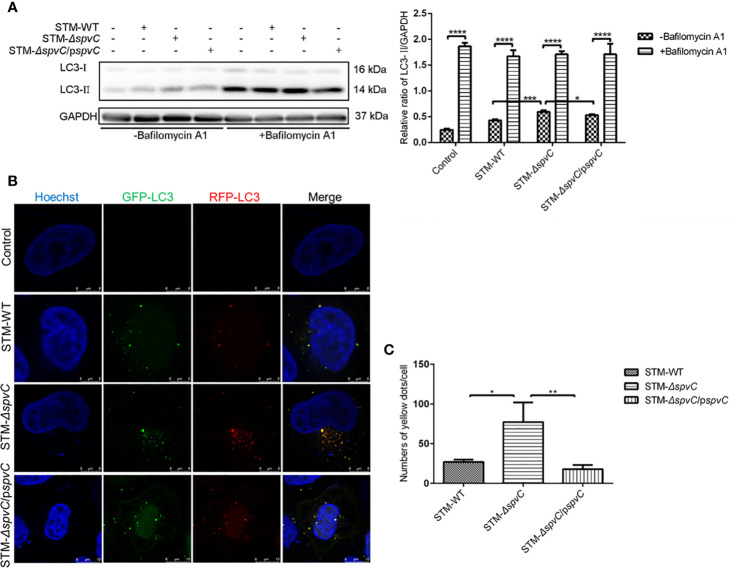
*SpvC* inhibits the formation of autophagosomes in host cells during *Salmonella* Typhimurium infection. **(A)** J774A.1 cells were infected with STM-WT, STM-*ΔspvC* or STM-*ΔspvC*/p*spvC* at an MOI of 10 after being pretreated with Bafilomycin A1. Cell lysates were analysed by western blot with specific antibodies to LC3 and the control GAPDH at 2 hpi. n = 3. **(B, C)** HeLa cells stability expressing mRFP-GFP-LC3 were used to establish an infection model. **(B)** LC3 puncta were visualized at 2 hpi by CLSM. Cell nucleus were labeled by Hoechst. **(C)** Punctate LC3 dots were measured by Image J. Number of punctate dots was enumerated in at least 100 cells. Scale bar, 5 µm. Data were compared by ANOVA. Values are expressed as the means ± S.D. and statistically significant differences are indicated. *P < 0.05; **P < 0.01; ***P < 0.001; ****P < 0.0001.

LC3-II, as the marker of autophagosomes, associates with both the outer and inner membranes of the autophagosomes. The increased protein level of LC3-II in **Figures 1A, B** indicates that *spvC* disturbs the number of autophagosomes in *S*. Typhimurium infected macrophages. Since autophagy is a dynamic process, the raised number of autophagosomes in STM-*ΔspvC* infected cells may represent either the increased formation of autophagosomes and/or the inhibition in autophagosomal maturation. Of interest, treatment of *S*. Typhimurium-infected macrophages with Bafilomycin A1, which blocks autophagosome-lysosome fusion, led to the accumulation of autophagosomes in all groups, but the magnitude of the increase was significantly lower in the STM-*ΔspvC* infected group than STM-WT or STM-*ΔspvC*/p*spvC* infected groups (**Figure 2A**). These data suggest that *spvC* negatively regulates autophagic activity and intervenes in the formation of autophagosomes in host cells. Furthermore, autophagic flux was morphologically monitored by mRFP-GFP-LC3. Autophagosomes and autolysosomes are labeled with yellow (RFP and GFP merged) and red (RFP only) puncta, respectively, since RFP exhibits more stable fluorescence in acidic compartments while GFP signal quenches for the low pH inside the lysosome (24). HeLa cells transfected with mRFP-GFP-LC3 were infected with different *S*. Typhimurium strains. More yellow LC3 puncta were visualized in STM-*ΔspvC* infected cells than those in STM-*ΔspvC* or STM-*ΔspvC*/p*spvC* infected cells at 2 hpi (**Figures 2B, C**). These results demonstrate that *spvC* inhibits autophagy by suppressing the formation of autophagosomes.

### *spvC* Phosphothreonine Lyase Activity Is Critical for Inhibiting Autophagy in *Salmonella* Typhimurium Infection

**Figure 3:**
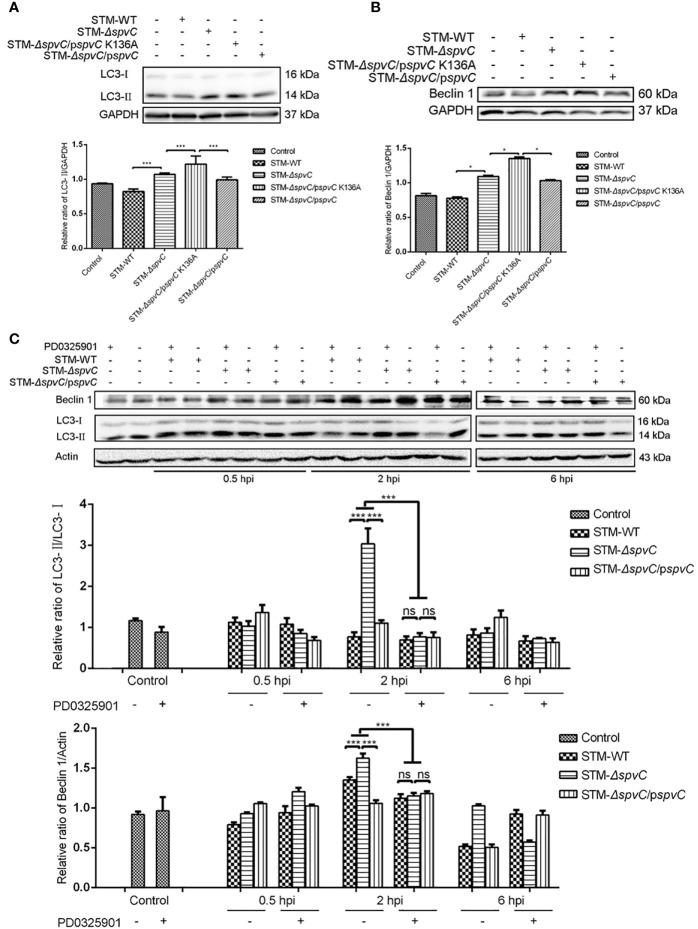
SpvC phosphothreonine lyase activity is critical for inhibiting autophagy in *Salmonella* Typhimurium infection. **(A, B)** J774A.1 cells were infected with STM-WT, STM-*ΔspvC*, STM-*ΔspvC*/p*spvC* or STM-*ΔspvC*/p*spvC* K136A at an MOI of 10 for 2 h. Cell lysates were analysed by western blot with specific antibodies to LC3 **(A)**, Beclin 1 **(B)** and the control GAPDH. **(C)** HeLa cells were infected with STM-WT, STM-*ΔspvC* or STM-*ΔspvC*/p*spvC* at an MOI of 100 after being pretreated with PD0325901. Cell lysates were analysed by western blot with specific antibodies to LC3, Beclin 1 and the control Actin at 0.5 hpi, 2 hpi and 6 hpi. Data were compared by ANOVA. Values are expressed as the means ± S.D., n = 3. Statistically significant differences are indicated. ***P < 0.001; *P < 0.01; ns, not significant.

Previous literature reported that SpvC is a *Salmonella* effector with phosphothreonine lyase activity on host MAPK (6). To investigate whether the enzymatic activity of SpvC is involved in its effect on autophagy, J774A.1 cells were co-cultured with STM-WT, STM-*ΔspvC*, site-directed mutant STM-*ΔspvC*/p*spvC* K136A which lacks phosphothreonine lyase activity and STM-*ΔspvC*/p*spvC*, respectively. As expected, both STM-*ΔspvC* and STM-*ΔspvC*/p*spvC* K136A gave rise to elevated levels of LC3-II in their infected macrophages compared with *S*. Typhimurium carrying *spvC* (**Figure 3A**). Concomitantly, levels of Beclin 1 were in line with the changing trend of LC3-II (**Figure 3B**). These results reveal that SpvC suppresses autophagy in macrophages through its phosphothreonine lyase activity.

ERK is an essential component in MAPK signaling pathway. To further elucidate the relationship between the effect of SpvC on autophagy and its phosphothreonine lyase activity on MAPK, HeLa cells were pretreated with ERK inhibitor PD0325901 before co-cultured with STM-WT, STM-*ΔspvC* or STM-*ΔspvC*/p*spvC*. In line with the results obtained in **Figure 1B**, significantly more LC3-II and Beclin 1 were determined in HeLa cells infected with STM-*ΔspvC* than those in cells infected with *S*. Typhimurium carrying *spvC* at 2 hpi. The conversion from LC3-I to LC3-II also correlates well with the number of autophagosomes (24). PD0325901 only decreased the expression of LC3-II and Beclin 1 in STM-*ΔspvC* infected cells rather than those infected with STM-WT or STM-*ΔspvC*/p*spvC*. Additionally, there was no significant difference of the expression of LC3-II and Beclin 1 among STM-WT, STM-*ΔspvC* or STM-*ΔspvC*/p*spvC* infected cells after PD0325901 treatment, which indicates that ablation of ERK signaling pathway virtually eliminates the inhibition of *spvC* on autophagy (**Figure 3C**). Collectively, these data indicate that phosphothreonine lyase activity of SpvC is required to inhibit the formation of autophagosomes.

### *spvC* Down-Regulates NLRP3 and NLRC4 in an Autophagy Related Manner

**Figure 4:**
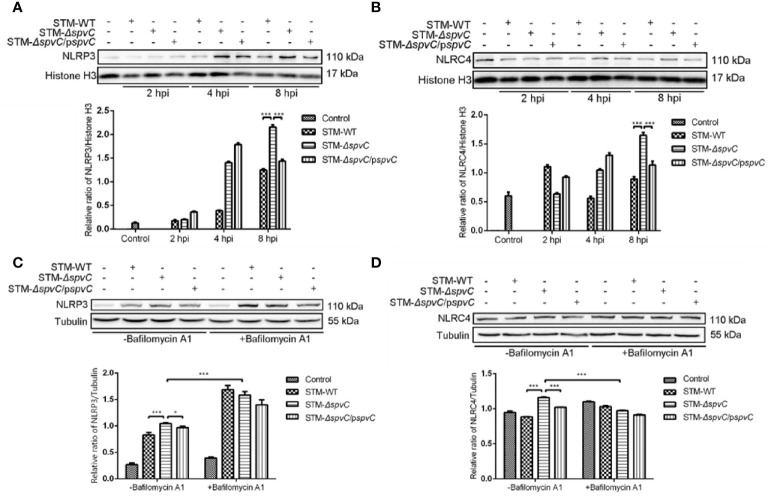
*spvC* down-regulates NLRP3 and NLRC4 in an autophagy related manner. **(A, B)** J774A.1 cells were infected with STM-WT, STM-*ΔspvC* and STM-*ΔspvC*/p*spvC* at an MOI of 10. Cell lysates were analysed by western blot with specific antibodies to NLRP3 **(A)**, NLRC4 **(B)** and the control Histone H3 at 2 hpi, 4 hpi and 8 hpi. **(C, D)** J774A.1 cells were infected with STM-WT, STM-*ΔspvC* and STM-*ΔspvC*/p*spvC* at an MOI of 10 at 8 hpi after being pretreated with Bafilomycin A1. Cell lysates were analysed by western blot with specific antibodies to NLRP3 **(C)**, NLRC4 **(D)** and the control Tubulin. Data were compared by ANOVA. Values are expressed as the means ± S.D., n = 3. Statistically significant differences are indicated. ***P < 0.001; *P < 0.01.

Previous studies have reported that SpvC exerts as an anti-inflammatory effector in systemic infection of *Salmonella* (5). Our earlier research has showed that *spvC* inhibits pyroptosis of host cells and it could also modulate NLRP3 and NLRC4-associated inflammatory response against *S*. Typhimurium. Of note, various literatures suggest that autophagy, a cellular waste removal and rejuvenation process, serves a crucial role as a macrophage-intrinsic negative regulator of NLRP3 inflammasome (25). In order to explore whether the autophagic response contributed to the effect of *spvC* on NLRP3 and NLRC4, we first co-cultured macrophages J774A.1 with STM-WT, STM-*ΔspvC*, or STM-*ΔspvC*/p*spvC* to investigate the dynamic function of *spvC* on NLRP3 and NLRC4. Western blot analysis showed the increasing evidence of NLRP3 and NLRC4 at 8 hpi due to the absence of *spvC* (**Figures 4A, B**), which suggests that *spvC* down-regulates NLRP3 and NLRC4.

Next, we generated the infection model pretreated with Bafilomycin A1. As previously mentioned, western blot analysis exhibited that in the absence of Bafilomycin A1, the levels of NLRP3 and NLRC4 in cells infected with STM-*ΔspvC* were significantly higher than that in cells infected with *Salmonella* carrying *spvC*. After Bafilomycin A1 treatment to inhibit autophagosome-lysosome fusion, the levels of NLRP3 and NLRC4 significantly changed though differences among three groups were still observed (**Figures 4C, D**). The results indicate that the effect of *spvC* on NLRP3 and NLRC4 is closely related to autophagy, but other factors are also involved in this process.

## Discussion

*S*. Typhimurium is not only a leading cause of human morbidity and mortality worldwide, but also a model pathogen for investigating the mechanisms of host-bacterium interactions (26). It is well known that macrophages, the professional phagocytes in host innate immune system, play a pivotal role in the clearance of *S*. Typhimurium. Autophagy is an important component of the innate immune system in host anti-bacterial defense, which is known to target a population of *Salmonella* for degradation and restrict *Salmonella* replication (27, 28). Beclin 1 interacts with several cofactors (*e.g.*, Atg14L, HMGB1, IP3R and PINK) to promote the formation of Beclin 1-Vps34-Vps15 core complexes, thereby inducing autophagy (9). LC3, a mammalian homolog of yeast Atg8, is known to serve as a widely used marker for autophagosomes. To assess a possible correlation between autophagy and *spvC*, we extended these studies by monitoring the time course of autophagy in macrophages during 24 h. Data showed that *spvC* restrains autophagy at the early stage of infection (2 hpi). Concomitantly, much more bacteria were counted in macrophages infected with *S*. Typhimurium carrying *spvC* at 2 hpi and 6 hpi, suggesting that *spvC* gene restricts elimination of pathogens in host cells which may related to autophagy.

However, some pathogens have evolved complex escape mechanisms of autophagy. We have previously shown that *spvB* blocks initial stage of autophagy and enhanced intracellular bacterial survival (29). In this study, we pretreated the infection model with Bafilomycin A1 which inhibits autophagosome-lysosome fusion, and results showed that *spvC* blocks the formation of autophagosomes. It is now appreciated that the devoured *Salmonell*a can survive after internalization into professional phagocytes (*e.g.* macrophages and neutrophils) and nonprofessional cells (*e.g.* epithelial cells) (30). Consistent with this, morphologically tracked autophagosomes (yellow puncta) and autolysosomes (red puncta) with mRFP-GFP-LC3 tandem construct indicates that *S*. Typhimurium harboring *spvC* inhibits the formation of autophagosomes.

As mentioned above, SpvC is a phosphothreonine lyase which exerts anti-inflammatory effects by inactivating dual-phosphorylated MAPK through beta elimination (31). A site-directed mutant STM-*ΔspvC*/p*ΔspvC* K136A which lacks phosphothreonine lyase activity was constructed. We found that the enzymatic activity of SpvC contributes to down-regulation of autophagy in macrophages. To date, study showed that *S*. Typhimurium *spvC* alleviated phospho-ERK1/2 expression in the villus epithelial cells and lamina propria of caeca, but no significant difference in phospho-p38 or phospho-JNK levels in the caeca infected with all strains (5). Based on this, HeLa cells were pretreated with ERK inhibitor PD0325901 before co-cultured with *S*. Typhimurium. We demonstrated that *spvC* affects the formation of autophagosomes in an ERK dependent manner. Besides we have proved that *spvC* inactivates phospho-ERK1/2, phospho-JNK and phospho-p38, leading to the interference of NLRP3 and NLRC4 in *S*. Typhimurium infected macrophages J774A.1 (18). Given all this, whether JNK and p38 are involved in *spvC* suppressed autophagy remains to be fully elucidated.

Several studies employing diverse bacterial species have highlighted the tactical interplay between autophagy and NLRP3 or NLRC4 inflammasomes. Therefore, macrophages J774A.1, applied in inflammation related research, were used to firstly valid that *spvC* down-regulates NLRP3 and NLRC4 at 8 hpi. Activation of NLRP3 inflammasome involves in damage to the mitochondria and the increased production of reactive oxygen species (ROS) (32). Autophagy plays a role in the removal of misfolded proteins, and the clearance of damaged mitochondria and ROS (33). Notably, we demonstrated that alleviated autophagosome formation is closely related to the effect of *spvC* on NLRP3. On the other hand, type ι interferon-dependent host response performs a negative feedback that represses expression of NLRC4 during *Salmonella* infection (34). Besides, we have reported that *Salmonella spv* locus could affect type ι interferon response *via* inhibiting autophagy in macrophages (35). Thus, we speculate that *spvC*-inhibited autophagy may be related to NLRC4. Indeed, experimental evidence reveals that the inhibition of autophagosome formation by *spvC* interferes with the level of NLRC4. Furthermore, MAPK can transmit signals from the cell membrane to the nucleus, which may provide the first signal for transcription of inflammasomes (36). This pathway is independent on the effect of autophagy on NLRP3 and NLRC4. Hence, repression of NLRP3 and NLRC4 by *spvC* contributes to the alleviation of pyroptosis, subsequently promotes bacterial dissemination in mice (18). Therefore, the role of NLRP3 and NLRC4 regulated by *spvC* to drive cell fate decisions between autophagy and pytoptosis in *Salmonella* infection deserves further investigation.

Taken together, we identify a novel contribution of the *spvC* gene to the pathogenesis of *Salmonella via* impairing the formation of autophagosome, thereby interfering with protein levels of NLRP3 and NLRC4. These findings have important implications for understanding the intricate evolutionary adaptations that shape host-pathogen cross-talk.

## Data Availability Statement

The original contributions presented in the study are included in the article/supplementary material. Further inquiries can be directed to the corresponding author.

## Ethics Statement

This study was approved by Soochow University Institutional Review Board.

## Author Contributions

LTZ, YL, and SW designed the research and wrote the manuscript. LTZ, YL, SG, HY, LLZ, and CW performed the research and conducted the data analysis. YL, RH, and SW supervised the project and edited the manuscript. All authors contributed to the article and approved the submitted version.

## Funding

The research leading to these results has received funding from the Natural Science Foundation of China (No. 31970132, No. 81971899), the Suzhou Municipal Science and Technology Foundation (SYS2019031), and the Priority Academic Program Development of Jiangsu Higher Education Institutions (PAPD).

## Conflict of Interest

The authors declare that the research was conducted in the absence of any commercial or financial relationships that could be construed as a potential conflict of interest.
